# Ultrabright near-infrared light

**DOI:** 10.1038/s41377-024-01416-2

**Published:** 2024-03-13

**Authors:** Andries Meijerink

**Affiliations:** Debye Institute for Nanomaterials Science, Utrecht University, Princetonplein 1, 3584 CC Utrecht, The Netherlands

**Keywords:** Nanoparticles, Diode lasers

*Nature Photonics* (2024)

10.1038/s41566-024-01400-7

The recent race for bright and broadband near-infrared (NIR) light sources has focused on new luminescent materials (phosphors) for efficient conversion of blue light emitting diode (LED) emission to broadband NIR. For extreme brightness, blue laser diodes (LDs) are superior to LEDs as they maintain efficiency at high current density. However, so far NIR phosphors suffered from severe efficiency droop at these high pumping powers. The team of Zhiguo Xia from South China University of Technology reported a record high 6 Watt NIR output from LD-pumped MgO:Cr^3+^ translucent ceramics emitting around 810 nm. Interestingly, MgO:Cr^3+^ is an old phosphor, extensively studied in the past century. The success is based in spark plasma sintering of translucent MgO:Cr^3+^ ceramics using nanosilica as flux, incorporated at grain boundaries. The refractive index contrast between MgO and SiO_2_ induces scattering of blue light, increasing light absorption, and the dense ceramic has excellent thermal conductivity, reducing thermal quenching of NIR emission. The new scheme using NIR-emitting translucent ceramics for laser-driven NIR light sources will propel photonics applications in night vision, biomedical imaging and sensing.

